# A long-read–based *de novo* assembly of *Magallana bilineata* for improved tropical oyster aquaculture

**DOI:** 10.1093/g3journal/jkaf242

**Published:** 2025-10-19

**Authors:** Matthew A Campbell, Luke W Silver, Nirooparaj Balachandran, Erandi Pathirana, Cara Jeffrey, Monal Lal, Wayne A O’Connor, Carolyn J Hogg, Joy A Becker

**Affiliations:** School of Life and Environmental Sciences, The University of Sydney, Sydney, NSW 2006, Australia; School of Life and Environmental Sciences, The University of Sydney, Sydney, NSW 2006, Australia; Australian Research Council Centre of Excellence for Innovations in Peptide and Protein Science, The University of Sydney, Sydney, NSW 2006, Australia; National Aquaculture Development Authority, Ministry of Fisheries and Aquatic Resources Development, Kilinochchi 44000, Sri Lanka; Faculty of Urban and Aquatic Bioresources, The University of Sri Jayewardenepura, Nugegoda 10250, Sri Lanka; School of Life and Environmental Sciences, The University of Sydney, Sydney, NSW 2006, Australia; School of Science, Technology & Engineering, University of the Sunshine Coast, Sunshine Coast, QLD 4556, Australia; Discipline of Marine Studies, School of Agriculture, Geography, Environment, Ocean and Natural Sciences, The University of the South Pacific, Lower Laucala Campus, Suva 0000, Fiji; New South Wales Department of Primary Industries and Rural Development, Port Stephens Fisheries Institute, Taylors Beach, NSW 2316, Australia; School of Life and Environmental Sciences, The University of Sydney, Sydney, NSW 2006, Australia; Australian Research Council Centre of Excellence for Innovations in Peptide and Protein Science, The University of Sydney, Sydney, NSW 2006, Australia; School of Life and Environmental Sciences, The University of Sydney, Sydney, NSW 2006, Australia

**Keywords:** black scar oyster, *Crassostrea*, Fiji, Indian backwater oyster, introduced species, *Magallana*, marine policy, Sri Lanka, translocations, computational scaffolding, genome assembly

## Abstract

True oysters, molluscs in the family Ostreidae, are important species in fisheries and aquaculture. As such, genome-enabled research can improve these industries and the conservation of these species. The tropical rock oyster *Magallana bilineata* (known as the black scar oyster or Indian backwater oyster) is naturally distributed in the tropical Indo-Pacific Ocean excluding Australia and is intensively cultured in India and the Philippines. It is also an aquaculture species in Sri Lanka with potential for much greater cultivation. We present the first reference genome for *M. bilineata* sourced from a Sri Lankan individual along with genetic variants that can be used in tool development for questions of molecular ecology and evolution as well as in breeding and commercial applications. Long-read PacBio data from a single *M. bilineata* were assembled following the Vertebrate Genomes Project workflow on the Galaxy Australia platform. A primary assembly composed of 105 contigs that is 551.94 Mbp in size was produced. The assembly N50 is 13.42 Mb and has a BUSCO completeness score of 98.1%. As collection and transport conditions challenged transcriptomic as well as scaffolding data generation, these approaches were undertaken computationally. Genetic variants in the form of SNPs from 90 individuals representing three naturally occurring populations in Sri Lanka and a fourth introduced population in Fiji was generated through DArTseq and a set of 3,115 SNPs produced after filtering. Combined, we present the first known genome assembly and the first genome-wide SNP data from *M. bilineata*, both of which have diverse applications for conservation and aquaculture.

## Introduction

**Fig. 1.**
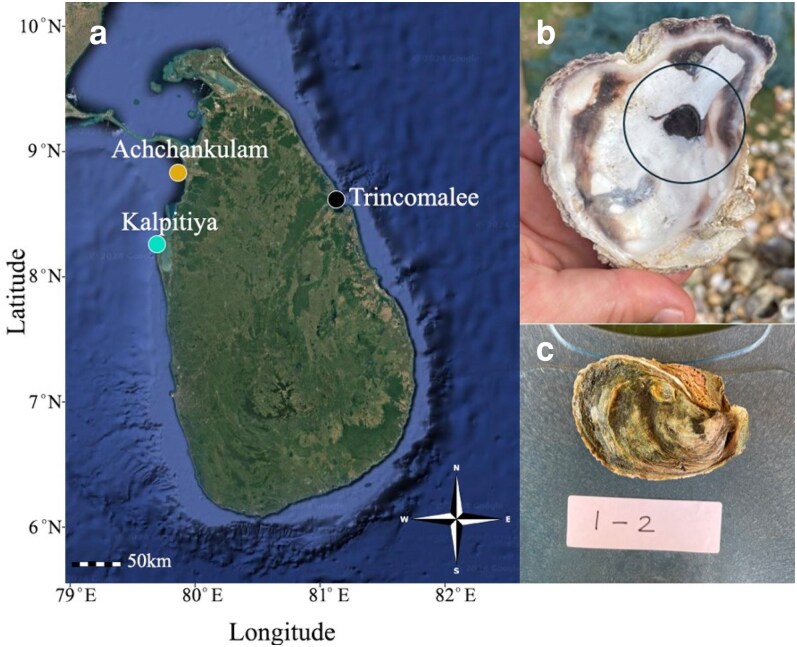
a) Location of *Magallana bilineata* sampling locations in Sri Lanka, b) photo of the dorsal valve of *M. bilineata* oyster noting the characteristic black scar, and c) photo of the oyster collected to build the reference genome (117 mm × 70 mm × 43 mm, total weight 183.3 g, meat weight 39.5 g).

Tropical rock oysters are an emerging aquaculture industry supporting local food security and rural livelihoods in developing countries. Farming in many areas is now moving toward large scale commercial production (Nowland et al. 2019). Several genera and numerous species of rock oysters are found in the tropics, with *Magallana bilineata* (Röding, 1798), a species of particular importance to aquaculture (Fig. 1). It is widely distributed in the tropical Indo-Pacific Ocean and, although not considered a native species of Australia or Fiji (Willan et al. 2021), can now be found in these regions. Common names for this species include the Indian backwater oyster or black scar oyster. Other scientific names that have been used recently, but are synonyms (Willan et al. 2021), are *Magallana iredalei* (Faustino, 1932) and *Magallana madrasensis* (Preston, 1916). *Magallana bilineata* is the most-commonly farmed oyster along the Indian coasts with industry development promoted by government programs that established successful family-led and women-led collectives for inclusive economic growth (Kripa et al. 2004; Suja et al. 2020). The production of *M. bilineata* emerged in the late 1990s in India and grew in the early 2000s to average ∼1,300 tonnes annually from 2000 to 2010 and has averaged 4,200 tonnes annually from 2011 to 2022 (FAO, 2024). Tropical rock oysters are considered a low-cost subsistence food that is low in calories and high in essential nutrients such as omega-3 fatty acids, iron, selenium, and vitamins D and B12 (Wright et al. 2018). *Magallana bilineata* is commonly found in large quantities in lagoons around Sri Lanka with wild collection and some attempts at farming to support local tourism (Subasinghe et al. 2021).

Taxonomic confusion is prevalent in tropical rock oysters, potentially slowing industry progress and allowing unknown introductions (Nowland et al. 2019; Sigwart et al. 2021). *Magallana bilineata* has a non-native distribution in tropical Australia (Willan et al. 2021) and was introduced to Fiji in the mid-1970s from the Philippines. In Fiji, *M. bilineata* has since become both a cultured and wild-harvested species (Ritchie, 1975; Kinch et al. 2019). Over the past 2 decades, Sri Lanka has seen attempts to establish tropical oyster farms using wild spat collection to supply local tourism markets. Production of *M. bilineata* in Sri Lanka peaked at 27 tonnes in 2018 (FAO, 2024) but dropped to essentially 0 tonnes annually in 2020 following terrorist bombings and the COVID-19 pandemic. Currently, there is one operational farm in Sri Lanka on the northwest coast producing less than 1 tonne annually. In contrast, the Indian *M. bilineata* industry yielded 4,600 tonnes in 2022, valued at $5.1 million USD (FAO 2024). The production of *M. bilineata* in the Philippines, formerly *M. iredalei* now classified under *M. bilineata*, has grown to average 30,500 tonnes annually in the 10-year span of 2013 to 2022 (FAO, 2024). Sri Lanka's tropical rock oyster aquaculture industry lags that of other Asian countries, particularly India, where the industry is well-established and growing despite their proximity and similar oceanic resources and environmental conditions.

According to the latest FAO food balance sheet, Sri Lanka is classified as a high-priority country due to the number of undernourished people (FAO et al. 2024). Since 2020, the cost of an affordable healthy diet has risen by 44%, and by 2022, 9 million Sri Lankans, i.e. 40% of the population, could not afford such a diet (FAO et al. 2024). As highlighted by Nowland et al. (2019), tropical oysters, due to their ease of culture, offer a cost-effective food source to combat hunger and malnutrition while also providing economic opportunity, particularly for women in rural areas. This is especially pertinent in Sri Lanka, where decades of civil conflict have resulted in a large population of widows facing challenges in accessing employment (Sarvananthan 2015).

We sequenced and assembled the first reference genome for *M. bilineata* to enable genome-based research to enhance global aquaculture and conservation for this species. We also generated a data set of genome-wide genetic polymorphisms (SNPs) placed on the reference genome to aid in tool development. Genomic tools can inform fundamental biological questions about adaptation and inheritance with practical applications for aquaculture development (e.g. breeding programs). Using the SNP data, we examine the divergence of Fijian and Sri Lankan populations of *M. bilineata* and tested for population genetic structure within Sri Lanka. This genomic information will support genetic management of the species, facilitating industry development and informing evidence-based translocation policies to accelerate aquaculture activities.

## Methods and materials

### Genome assembly

Six adult *M. bilineata* were collected from an oyster farm in Kalpitiya Lagoon, Sri Lanka, on 2024 February 24 (Fig. 1). The farm collects wild spat from nearby coastal areas for on-growing to market size. From each oyster, a small piece of gill, mantle, and adductor muscle (∼50 mg each) was excised, placed in separate tubes containing RNAlater (4 mL each) and then stored at 4 °C. Dissection tools and work surfaces were disinfected using sodium hypochlorite (200 ppm for 10 min), rinsed in fresh water, and sprayed with 70% ethanol immediately prior to use. New sets of gloves and sterile disposable scalpel blades were changed for each oyster dissection. For importation into Australia, the RNAlater was decanted and the samples preserved in molecular grade ethanol (topped up to 5 mL). After arrival in Australia (approximately 36 h), the ethanol was decanted and replaced with RNAlater and frozen at −80 °C until extraction. High molecular weight DNA was extracted from ∼25 mg of tissues with a Nanobind extraction kit (Circulomics Inc., Baltimore, MD, USA) with no modifications to the protocol. Highest quality resulted from gill tissues and DNA from a single individual was sequenced. Library preparation of HiFi SMRTbell libraries was performed with a SMRTbell Express Template Prep Kit 2.0 (Pacific Biosciences of California Inc., Menlo Park, CA, USA) and sequenced on a PacBio Revio machine with one SMRT cell at the Australian Genome Research Facility (AGRF, Queensland).

To generate the *M. bilineata* reference genome, the vertebrate genomes project (VGP) assembly pipeline was followed in the solo mode with only PacBio HiFi reads using the Galaxy Australia interface (Batut et al. 2018; The Galaxy Community 2022; Hiltemann et al. 2023; Lariviere et al. 2024). Briefly, HiFi reads were quality trimmed and reads containing adapters were removed using Cutadapt v4.6 (Martin 2011), and genome size and *k*-mers were estimated using Meryl v1.3 (Rhie et al. 2020) and GenomeScope v2.0 (Ranallo-Benavidez et al. 2020). Genome assembly was performed using hifiasm in the solo mode with the purging level set to light (Cheng et al. 2021) using HiFi reads to assemble contigs. Assembly quality of the primary and alternate assemblies was assessed using gfastats v1.3.6 (Formenti et al. 2022) and BUSCO v5.5.0 with the mollusca_odb10 (*N* = 5295) lineages (Simão et al. 2015). BUSCO revealed an incomplete alternate assembly and duplications in the primary assembly, so purging was undertaken using purge_dups to remove duplicate contigs from the primary assembly and place them in the alternate assembly. A second round of purge_dups was run on the alternate assembly.

Final genome analysis was conducted using BUSCO v5.5.0 with the mollusca_odb10 lineage, Fasta Statistics tools provided in Galaxy Australia and Merqury v1.3 (Rhie et al. 2020). Repetitive elements of the genome were identified and masked using RepeatModeler v2.0.4 and RepeatMasker v4.1.5 (Flynn et al. 2020). Annotation was conducted with the MAKER genome annotation pipeline v3.01.04 (Cantarel et al. 2008; Holt and Yandell 2011). Generic control files were generated with MAKER (-CTL option), and as the quality of RNA preserved was insufficient for transcriptomics, a set of proteins in amino acid sequence format from the *M. gigas* (Pacific Oyster) reference genome (GCF_963853765.1; GCF_963853765.1_xbMagGiga1.1_protein.faa) were supplied as protein homology evidence to the MAKER annotation pipeline. Subsequently, protein-coding gene annotations were filtered based on an Annotation Edit Distance Evidence (AED; Eilbeck et al. 2009) of at least 0.50 and a minimum length of 50 amino acids to train the *ab initio* gene prediction program SNAP (Semi-HMM-based Nucleic Acid Parser; Korf 2004). A second round of MAKER was run pointing to the previously identified protein-coding genes and repeat sequences identified in the initial MAKER run and using SNAP gene models in prediction.

### Scaffolding

Sample collection in Sri Lanka poised several challenges for meeting the input requirements for the generation of scaffolding data such as HiC, including the unavailability of liquid nitrogen for flash freezing. As a result, we computationally scaffolded our assembly using the three available congeneric assemblies of *M. gigas* (GCF_963853765.1), *M. angulata* (GCF_025612915.1), and *M. hongkongensis* (GCA_015776775.1). All three of these species are from a clade of *Magallana* that split from the lineage containing *M. bilineata* approximately 52.5 million years ago (Kumar et al. 2022). None of the closer relatives of *M. bilineata* have high-quality genome assemblies publicly available (i.e. *M. dianbaiensis*, *M. iredalei*, and an undescribed taxon). The three reference genomes of *M. gigas*, *M. angulata*, and *M. hongkongensis* have 10 chromosomes in their genomes, and those were extracted from the entire assemblies of each reference species for computational scaffolding with RagTag v2.1.0 (Alonge et al. 2022). A computationally scaffolded version of the *M. bilineata* genome was created with each of the three congeneric reference genomes using the *scaffold* command of RagTag with other options set to defaults. We tested for alterations to BUSCO scoring by assessing the *de novo M. bilineata* and all three computationally scaffolded systems with BUSCO v6.0.0 and the mollusca_obd12 data set (Tegenfeldt et al. 2025).

To compare the three computationally scaffolded assemblies of *M. bilineata*, we aligned the *M. angulata* and the *M. hongkongensis* scaffolded assemblies of *M. bilineata* to the *M. gigas* scaffolded assembly with minimap2 v2.28-r1209 (Li, 2018, 2021). Following the program manual, we specified a scoring system of “asm5” for intraspecies alignment and allowed continuous alignments (did not use -r option). The first 12 columns of the output of the alignment were kept for further analyses. The alignment outputs were loaded into R v4.3.2 (R Core Team 2024), filtered, and visualized with functions of the tidyverse package v2.0.0 (Wickham et al. 2019). Alignments were filtered to remove alignments occurring due to overall similarity at small scales by filtering for a minimum length of 100,000 bp and a match quality score of greater than or equal to 60. Macrosyntenic patterns were characterized through visualization. We repeated this process with the three genomes used in scaffolding.

### Genome-wide SNP data

A small piece of adductor muscle was excised as above from 20 *M. bilineata* individuals from three locations (60 total) in Sri Lanka and preserved in molecular biology grade ethanol topped up to 5 mL total volume (Fig. 1). Sri Lankan locations were from three provinces/geographic regions of Sri Lanka, the Eastern Province (Trincomalee), the North-Western Province (Kalpitiya), and the Western Province (Achchankulam). An additional 30 individuals were sourced from Fiji (Vutia, Viti Levu, Fiji). Individual sample details are provided in Supplementary Table 1. Preserved tissues were sent to Diversity Arrays Technology (DArT Pty Ltd, Canberra, Australia) for DNA extraction and genotyping with the DArTseqTM methodology. This approach is a type of double-digest RAD sequencing technique, following methods from Georges et al. (2018) with a combination of *PstI* and *SphI* restriction enzymes. Resulting reads were aligned to the new reference genome for SNP calling and the resulting variants were supplied by DArT in a VCF-formatted file.

Data were imported into R for analysis with snpR v1.2.9.1 (Hemstrom and Jones 2023). Both individuals and SNP loci were filtered with the filter_snps function of snpR to a minimum minor allele frequency (MAF) of 0.05, presence of a SNP in at least 90% of individuals, and individuals were required to have 90% of SNPs present (maf = 0.05, min_ind = 0.9, and min_loci = 0.9). Linked SNPs were removed under the Burrow's Composite Linkage Disequilibrium method (LD_prune_r = 0.3, LD_prune_sigma = 10, LD_prune_method = “CLD”). Principal component (PC) analysis was conducted and sparse non-negative matrix factorization (SNMF) of this total data set using functions in snpR. The SNMF analysis calls the snmf function of the LEA package (Frichot and François 2015), and *K* = 2 to 6 genetic clusters were evaluated and compared through cross-entropy scores. Genetic structure was also assessed between sampling locations by calculating pairwise *F_ST_* using weighted means with the calc_pairwise_fst function of snpR. Subsequently, data were filtered to only Sri Lankan sampling locations and the same filtering, PC analysis, and clustering steps undertaken.

### Additional tests for population genetic structure

To further test for the presence of population genetic structure and potential adaptive genetic variation within Sri Lankan oysters, we applied the R package pcadapt v4.4.0 (Luu et al. 2017). We used the previously filtered SNPs with samples only from Sri Lankan sampling locations and followed the guidance of the pcadapt manual, first plotting a scree plot for up to 20 principal components to identify a steep curve associated with possible genetic structure. Following a lack of clear changes in percentage of variance across PCs, we tested for outlier loci along the first two PCs with both Benjamini and Hochberg and Bonferroni corrections at a desired significance level of 0.10.

Fine-scale genetic structure was explored across Sri Lankan samples at selectively neutral loci using the Netview R package (Neuditschko et al. 2012; Steinig et al. 2016). Netview R population networks were created by first generating a genetic distance matrix (shared allele 1-identity-by-state [IBS]) in the PLINK v1.07 toolset (Purcell et al. 2007). Multiple networks were then constructed using the IBS matrix by computation of the maximum number of nearest neighbors for each individual (Tsafrir et al. 2005; Neuditschko et al. 2012; Steinig et al. 2016), at a maximum number of nearest neighbor (mk-NN) values between 1 and 50, after which the optimal network for representation was selected based on cluster stability (Steinig et al. 2016). Individual networks were visualized and edited in the Cytoscape v2.8.3 package (Smoot et al. 2011).

A discriminant analysis of principal components (DAPC) was used to investigate genetic structure, implemented in the *R* package adegenet v1.4.2 (Jombart 2008; Jombart et al. 2010; Jombart and Ahmed 2011). An α-score optimization was used to refine the DAPC plot by informing the number of principal components to retain and the “find.clusters” function of *adegenet* used for identifying the optimal number of *k-*clusters using a Bayesian information criterion method (*k*-means clustering). In some circumstances, group memberships of a DAPC may be subject to overfitting the discriminant functions when too many principal components are retained, therefore the “compoplot” function of adegenet was also utilized to generate a STRUCTURE-like barplot of group membership probability. This method permitted elucidation of biologically defined group memberships based on retained discriminant functions only, instead of relying on *k-*means clustering alone (Jombart and Collins 2015).

### Population genetic statistics

We examined genetic diversity in terms of nucleotide diversity (*π*) to assess if Sri Lankan populations of *M. bilineata* sampled were largely similar. We also calculated Tajima's D (*D*) from Sri Lankan populations to assess if the values deviated among sampling locations within Sri Lanka. We calculated *π* with the cal_pi function and *D* with the calc_tajimas_d functions of snpR v1.2.9.1. For both statistics, the mean value across contigs of the *de novo* assembly was calculated and those used to create boxplots of each statistic for each Sri Lankan sampling location.

## Results and discussion

### Genome assembly

**Fig. 2.**
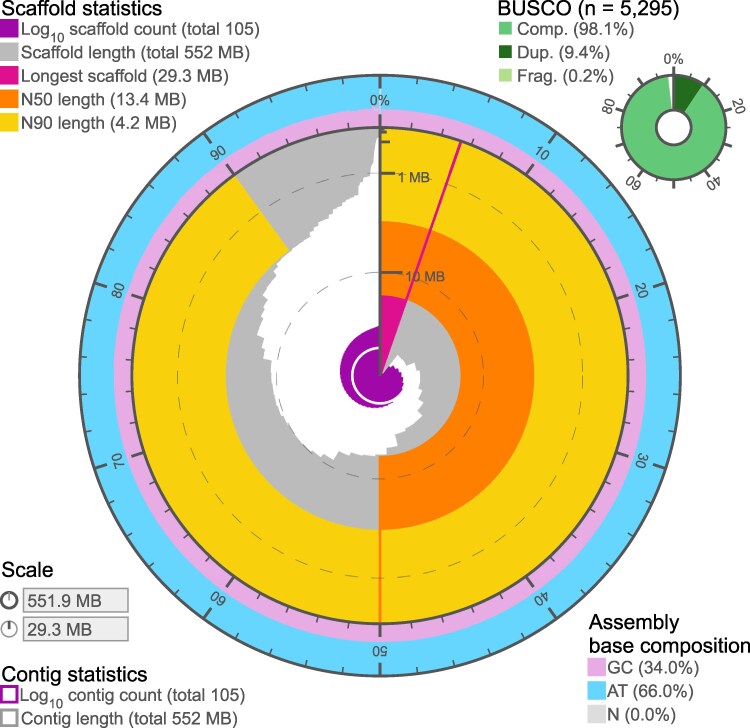
Visual overview of *Magallana bilineata* genome assembly metrics. Circular plot generated using assembly-stats (10.5281/zenodo.594927) depicting quality metrics presented in Table 1. The circle represents the full length of the assembly (551.9 Mb). Length related metrics are represented in the middle. The dark pink line shows the longest scaffold, followed by all other scaffolds in size-order moving clockwise shown in yellow. The purple spiral displays the cumulative scaffold count. The N50 and N90 values are represented in dark and light orange, respectively. The exterior pink and blue circles show mean, maximum, and minimum GC vs AT levels. BUSCOv5.5.0 (mollusca_odb10) scores are shown in the top right corner including complete (single and duplicated) (Comp.), duplicated (Dup.), and Fragmented (Frag.).

Sequencing with PacBio technology for reference genome assembly produced 4.1 million reads averaging 9,480 bp for a total of 39.2 Gbp of raw sequence data. The initial genome assembly resulted in a primary and alternate haplotype assembly for *M. bilineata.* The primary assembly contained 228 contigs and was 704.29 Mbp in size (Fig. 2), with the alternate assembly smaller and less contiguous with 1,374 contigs and 287.20 Mbp in size (Table 1). BUSCO quality assessment revealed a highly complete primary assembly, with 98.9% complete BUSCOs present; however, 39.3% of these were duplicated. Additionally, the alternate assembly was much less complete with only 59.8% complete BUSCOs. As a result of this, we undertook purging of the primary and alternate assemblies. Purging resulted in a much less duplicated primary assembly and more complete alternate assembly. The final primary assembly contains 105 contigs and is 551.94 Mbp in size with an N50 of 13.42 Mb, and the alternate assembly consists of 582 contigs and is 410.77 Mbp in size with N50 of 2.73 Mb (Table 1). BUSCO analysis also showed improvements compared to the unpurged assemblies, primary assembly with 98.1% complete BUSCOs with 9.4% duplicated and the alternate assembly 89.4% complete (Table 1). Finally, analysis of both alternate and primary assemblies with Merqury showed 99.41% of *k*-mers present across the two haplotypes and quality value score of 59.51 in the final assembly (Table 1). Repeat masking resulted in masking 41.1% of the genome, the majority of which are unclassified (33.86% of the genome) (Table 2). The first round of MAKER annotation generated 28,506 protein-coding genes from the alignment of the *M. gigas* protein sequences. Filtering for an AED score of at least 0.50 and length of 50 amino acids for training SNAP reduced this number to 22,739 genes. A second round of annotation with MAKER in conjunction with SNAP produced a total of 33,288 protein-coding genes (Supplementary File 2). Filtering for AED scores < 0.49 and a length of at least 50 amino acids produces a set of 28,685 genes with a mean length of 490 amino acids. There are 2 NCBI RefSeq assemblies for *Magallan*a that can provide some comparison. In the *M. gigas*, primary assembly is 564.0 Mb in size with 26,073 protein-coding genes, and in the *M. angulata* (Portuguese oyster, GCF_025612915.1), primary assembly is 624.3 Mb in size 29,810 protein-coding genes. The size and annotation we produced for *M. bilineata* are comparable to these other two species of *Magallana*.

**Table 1.** Summary statistics of the initial unpurged primary and alternate assemblies as well as the final primary and alternate assemblies for *Magallana bilineata*.

	Primary contigs	Alternate contigs	mCraBil1.alt.20240620	mCraBil1.pri.20240620
Methods	Primary haplotype assembled with hifiasm	Alternate haplotype assembled with hifiasm	Purged alternate assembly	Purged primary assembly
Number bases (Mb)	704.29	287.20	410.72	551.94
GC%	34.07	34.05	34.04	34.01
Gaps (%)	0	0	0	0
Contig L50	21	45	39	14
Contig L90	62	232	159	41
Contig N50 (Mb)	10.19	1.98	2.73	13.42
Contig N90 (Mb)	3.66	0.09	0.52	4.21
Number contigs	228	1374	582	105
Longest contig (Mb)	29.28	8.05	10.59	29.27
BUSCO mollusca_odb10 (*n*: 5295)	C: 98.9% [S: 59.6%, D: 39.3%], F: 0.2%, M: 0.9%	C: 59.8% [S: 58.3%, D: 1.5%], F: 0.3%, M: 39.9%	C: 89.4% [S: 88.7%, D: 0.7%], F: 0.4%, M: 10.2%	C: 98.1% [S: 88.7%, D: 9.4%], F: 0.2%, M: 1.7%
Merqury quality value			59.39	59.51
Merqury *k*-mer completeness			64.92%	79.00%

**Table 2.** Classification and number of repeats masked in the primary purged *Magallana bilineata* assembly.

	Number of elements	Length (bp)	% of sequence
Retroelements	59,503	21,956,308	3.98
SINEs	8,941	1,523,363	0.28
Penelope	7,230	1,681,206	0.30
LINEs	35,680	10,002,091	1.81
L2/CR1/Rex	3,911	783,982	0.14
R1/LOA/Jockey	1,021	119,008	0.02
R2/R4/NeSL	146	109,652	0.02
RTE/Bov-B	11,562	2,950,608	0.53
L1/CIN4	648	411,240	0.07
LTR elements	14,882	10,430,854	1.89
BEL/Pao	1,018	775,853	0.14
Ty1/Copia	743	244,945	0.04
Gypsy/DIRS1	10,225	7,777,284	1.41
DNA transposons	60,131	16,365,199	2.97
hobo-Activator	1,712	1,073,301	0.19
Tc1-IS630-Pogo	84,88	3,460,398	0.63
MULE-MuDR	667	246,801	0.04
Piggybac	832	184,518	0.03
Tourist/Harbinger	4,038	654,856	0.12
Rolling-circles	7,376	3,282,570	0.59
Unclassified	675,766	186,860,179	33.86
Total interspersed repeats		226,862,892	41.10
Small RNA	7,245	1,461,698	0.26
Satellites	442	175,034	0.03
Simple repeats	812	49,792	0.01

### Scaffolding

Computational scaffolding placed 65 to 68 of the contigs into chromosomes with RagTag (Table 3). This approach increased the mean length of sequences in the *M. bilineata* assembly from 5.26 Mbp to between 11.04 and 11.74 Mbp depending on the reference genome used. The maximum sequence length increased from ∼29 Mbp to ∼70 to 80 Mbp depending on the reference genome aligned to. The resulting N50 values also increased substantially from ∼13 Mbp to ∼53 to 57 Mbp and reduced the number of sequences in the N50 from 14 to 5. All these metrics indicate that the computationally scaffolding overall increased the contiguity of the *M. bilineata* genome. BUSCO scores declined from a 98.9% score for the de novo assembly to 98.1 to 98.4% for the computationally scaffolded assemblies. The proportion of duplicated BUSCO genes declined from 9.7% to 8.7 to 9.2%. However, the percentage missing did increase from 1.0% to 1.5 to 1.8%. No single computationally scaffolded genome appears to have significant improvements by this metric.

**Table 3.** Metrics of de novo assembly of *Magallana bilineata* and from computational scaffolding using *M. gigas*, *M. angulata*, and *M. hongkongensis* reference genomes.

	*M. bilineata* de novo	Scaffolded with *M. gigas*	Scaffolded with *M. angulata*	Scaffolded with *M. hongkongensis*
Placed contigs	-	66	68	65
Placed bp	-	540,321,084	545,295,692	537,280,775
Unplaced Contigs	-	39	37	40
Unplaced bp	-	11,619,108	6,644,500	14,659,417
Total bases	551,940,192	551,945,792	551,945,992	551,945,692
Mean sequence length	5,256,573	11,264,199	11,743,531	11,038,913
Max sequence length	29,274,846	72,195,338	79,965,720	69,966,113
N50	13,422,316	56,188,369	56,797,364	53,006,122
Number of sequences in N50	14	5	5	5
BUSCO mollusca_odb12 (*n*: 4421)	C: 98.9% [S: 89.2%, D: 9.7%], F: 0.1%, M: 1.0%	C: 98.4% [S: 89.2%, D: 9.2%], F: 0.1%, M: 1.5%	C: 98.1% [S: 89.4%, D: 8.7%], F: 0.1%, M: 1.8%	C: 98.1% [S: 89.1%, D: 9.0%], F: 0.1%, M: 1.8%

**Fig. 3.**
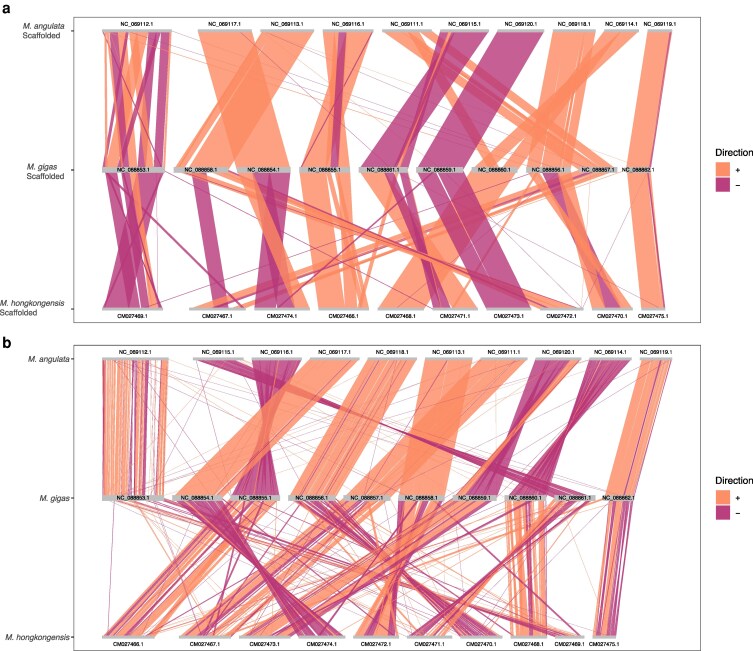
Macrosyntenic comparison of genome assemblies. a) The alternative computationally scaffolded versions of the *Magallana bilineata* genome. For each of 3 reference genomes used—*M.* a*ngulata*, *M. gigas*, and *M. hongkongensis—*the reference chromosome is indicated and reordered by size. b) The same macrosyntenic comparison between reference assemblies of *M. angulata*, *M*. *gigas*, and *M. hongkongensis.* Chromosome sequences are in order of accession numbers. Direction of the alignments is indicated by color.

The *M. angulata* and *M. gigas* scaffolded versions of the *M. bilineata* assembly are largely congruent with a high degree of collinearity, with the exception of the sequences aligned to reference chromosomes NC_069112.1 in *M. angulata* and *M. gigas* NC_0888531.1 (Fig. 3). This largest chromosome in both assemblies shows substantial differences in the order of contigs placed on that chromosome. Less collinearity occurs between the *M. gigas* scaffolded version of the *M. bilineata* assembly and the *M. hongkongensis* scaffolded version of the *M. bilineata* assembly in comparison. The largest chromosome of the *M. hongkongensis* scaffolded assembly, reference chromosome CM027469.1, while overall more contiguous with the *M. gigas* reference chromosome NC_0888531.1, also shows some large rearrangements.

In the current study, materials to collect and facilities to conduct data generation for scaffolding using materials obtained in Sri Lanka were not possible. These challenges are not unique to work undertaken across the globe, especially in the global south (Carneiro et al. 2025). We applied computational scaffolding (RagTag) to the *de novo* assembly of *M. bilineata* to place the genome into chromosomes to overcome this challenge computationally. The three alternative computationally scaffolded genomes are similar, but the *M. angulata* scaffolded assembly incorporates two more contigs than the *M. gigas* reference-based version resulting in an increase of ∼5 Mbp incorporated into chromosomes of the scaffolded assembly. The alternative scaffolding approaches (Fig. 3) are differentiated with respect to the largest chromosome (reference chromosomes NC_0888531.1, NC_069112.1, CM027469.1). Differences observed may be a result of either true differences underlying the assemblies of the three reference genomes, or artifacts of their construction. The three reference genomes used in computational scaffolding are all more closely related to each other than *M. bilineata*, with a time to most recent common ancestor of *M. bilineata* from *M. angulata*, *M. hongkongensis*, and *M. gigas* approximately 52.5 million years (Li et al. 2021; Kumar et al. 2022). *Magallana hongkongensis* is substantially diverged from *M. angulata* and *M. gigas* at ∼34 million years, with *M. angulata* and *M. gigas* being much more closely related at 3.7 million years of divergence. With hypothetically well-assembled reference genomes of similar quality among the three species used for computational scaffolding, the scaffolding of *M. bilineata* to *M. angulata* and *M. gigas* would produce the most similar results. This does appear to be the case with more macrosyntenic differences observed between the *M. gigas* scaffolded genome and the *M. hongkongensis* scaffolded genome (Fig. 3). Nonetheless, it does appear the composition of the main chromosomes of *M. bilineata* can be inferred if large scale conservation within *Magallana* in terms of chromosomal composition is assumed. Given that the technical and practical limits for genomics data generation worldwide may remain in place, computational scaffolding may be an important intermediate step for the generation of some reference genomes.

### Genome-wide SNP data

**Fig. 4.**
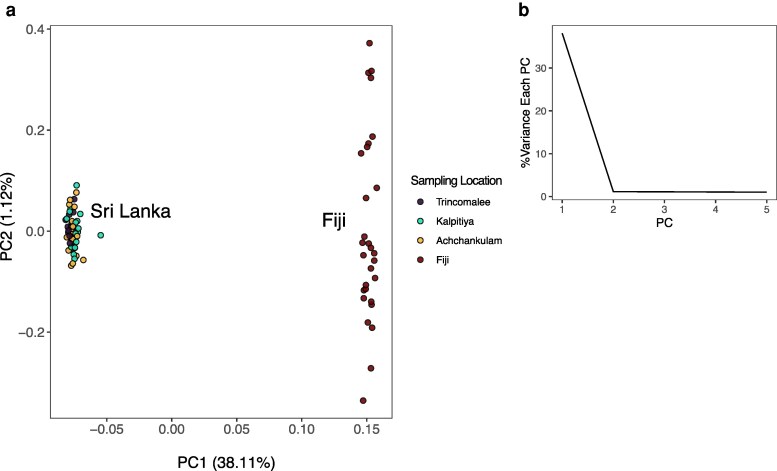
Prinicpal component (PC) analysis of 87 samples and 3,046 SNPs. a) PC1 (38.11% of variance) and PC2 (1.12% of variance). b) A scree plot of variation across the first five PCs.

Across all 90 samples, a total of 27,332 SNPs were identified (VCF file provided as Supplementary File 1). Filtering reduced the total to 87 individuals, with the loss of two individuals from Trincomalee and one from Fiji. The number of SNPs after filtering and pruning for linkage disequilibrium (LD) was 3,046. Variation in the SNPs separates all Sri Lankan sampling locations from the Fiji sampling location (38.11% of variation) with the second PC (1.12% of variation) corresponding to variation within the Fijian samples (Fig. 4a). A scree plot and examination of variation across PCs indicates that the first axis should be the primary consideration (Fig. 4b). Population structure testing with SNMF also supports two distinct groupings (Supplementary Fig. 1). Values of *F_ST_* ranged from 0.002 to 0.485 (Supplementary Fig. 2), with values of *F_ST_* within Sri Lanka the lowest (0.003 to 0.005). The Fijian samples were highly differentiated from Sri Lanka (*F_ST_* 0.476 to 0.485). Filtering SNPs and individuals from only Sri Lankan samples, removed two samples from Trincomalee (*n* = 58 total) and resulted in 2,913 SNPs. There was no clear evidence of population genetic structure in Sri Lanka apparent in *F_ST_* values (Supplementary Fig. 3).

### Additional tests for population genetic structure and population genetic statistics

The use of pcadapt to test for potential genetic structure and possible adaptive loci across 2,913 SNPs and *n* = 58 Sri Lankan samples did not identify and meaningful population genetic structure in the screeplot nor did the tests for outlier loci along the first two PCs return any variants (results not shown). The population network analysis with Netview *R* does not support population genetic structuring (Supplementary Fig. 4) nor does DAPC (Supplementary Figs. 5 and 6). Comparable SNP-based data sets of other oysters have found similar results. Lal et al. (2016) examined black-lipped pearl oysters *Pinctada margaritifera* from 11 sites in Fiji with 4,123 SNPs and found *F_ST_* values of 0.002 to 0.005 and concluded on a lack of genetic structure. Examination of Sydney rock oysters, *Saccostrea glomerata,* across 25 sites in eastern Australian estuaries with 3,400 SNPs found *F_ST_* values to be less than 0.0038 and also a lack of genetic structure (O’Hare et al. 2021). It is important to note that our data was based on a subset of variation of the genome (DArTseq) and that key adaptive variants may not be detected by this methodology (Lowry et al. 2017). However, the same basic data type we applied (double-digest RADseq) in *Crassostrea virginica* does resolve adaptive differentiation and population genetic structuring and influences of captive propagation on the genetic makeup of populations in different study areas (Hornick and Plough 2021; Strickland and Brown 2024).

Values of *π* from Sri Lankan populations of *M. bilineata* sampled are very similar, from 0.29 to 0.30 (Supplementary Fig. 7). The measures of *D* are all positive and largely similar, from 0.76 to 0.88, with the lowest *D* from Trincomalee and the highest from Achchankulam. These values of *D* are consistent with rare alleles being scarce, which may be attributed to balancing selection, or population contraction (Tajima 1989). These statistics indicate that the overall diversity and demographic history are consistent across Sri Lankan sampling locations sampled in this study in line with the observation that they appear to be a single genetic population.

## Conclusions

We provide the first assembly of *M. bilineata*, and it is highly contiguous and begins an era of genome-enabled research for this species. Genomic variation is substantial between Sri Lankan and Fijian sampling locations in this study, 38.11% of variation in PC analysis (Fig. 4). Fijian samples were introduced from the Philippines and may be considered representative of *M. iredalei*. The taxonomy of tropical oysters is in flux and contentious (Sigwart et al. 2021), and *M. iredalei* has been considered a synonym of *M. bilineata* (Willan et al. 2021). Our evidence supports the separation of *M. bilineata* and *M. iredalei* based on the magnitude of this differentiation and provides nuclear markers that can be used to further evaluate the taxonomic reality of *M. iredalei* as has been advocated by Sigwart et al. (2021). Within Sri Lanka, we do not find support for genetic structure of *M. bilineata*, with additional validation of this finding important for informing genetic policy within Sri Lanka. The outcomes from this study provide foundational knowledge to develop within Sri Lanka translocation policy for the movement of oyster stock to support aquaculture development.

## Supplementary Material

jkaf242_Supplementary_Data

## Data Availability

Genotypes are provided as Supplementary File 1 in VCF format. The reference genome and raw sequence data for assembly are available under NCBI BioProject PRJNA1181550 with the genome reported in this manuscript version JBLMKS010000000. Raw DArTseq data are available under NCBI BioProject PRJNA1181550. Full annotation output from MAKER and computationally scaffolded genomes are available at https://doi.org/10.25387/g3.29095958. Supplemental material available at G3 online.
