# Supplementary material for: A long-read–based de novo assembly of Magallana bilineata for improved tropical oyster aquaculture
Source: G3 (Bethesda). 2025 Oct 19;15(12):jkaf242. doi: 10.1093/g3journal/jkaf242 (PMC12693617; doi:10.1093/g3journal/jkaf242)
Supplement: jkaf242_Supplementary_Data [file jkaf242_supplementary_data.zip › Supplemental_Figure_and_File_Legends_G3-2025-406155.docx]

Supplemental Figure S1: Admixture analysis of 87 samples and 3,046 SNPs with sparse non-negative matrix factorization (SNMF). Panel A shows individual membership probabilities across sampling locations for *K* = 2..6. Panel B shows cross entropy scores for *K* = 2 to 6.

Supplemental Figure S2: Evaluation of population genetic structure from 58 Sri Lankan Black Scar Oysters *Magallana bilineata* from three sampling locations and 2,913 SNPs. Panel A depicts Principal Component (PC) analysis of the first two PCs, PC1 with 2.36% of variation and PC2 with 2.29% of variation. Panel B shows the percent of variation explained across the first five PCs. Panel C shows individual membership proportions for *K* = 2 to6 genetic clusters from sparse non-negative matrix factorization (SNMF). Panel D shows cross entropy scores for *K* = 2 to 6 genetic clusters in the SNMF analysis.

Supplemental Figure S3: Pairwise *F_ST_* calculated between sampling locations of *Magallana bilineata* included in this study from 87 individuals and 3,046 genome-wide SNPs.

Supplemental Figure S4: High-resolution population network of genetic structure in Sri Lankan *Magallana bilineata* sampled at three sites generated using Netview *R* (Steinig et al., 2016)*.* The network is shown at a maximum number of nearest neighbour (mk-NN) threshold of 40, using 2,913 selectively-neutral SNPs and 58 individuals. Each dot represents a single individual and sampling location colour assignments are as follows: Achchankulam: gold, Kalpitiya: aquamarine and Trincomalee: dark purple.

Supplemental Figure S5: Discriminant Analysis of Principal Components (DAPC) α-score optimised scatterplot of Sri Lankan *M. bilineata* genetic structure using 2,913 selectively-neutral SNPs and 58 individuals. Oysters were sampled at three locations and sampling location colour assignments are as follows: Achchankulam: gold, Kalpitiya: aquamarine and Trincomalee: dark purple.

Supplemental Figure S6: Discriminant Analysis of Principal Components (DAPC) individual group membership barplot (‘compoplot’) for Sri Lankan *M. bilineata* sampled at three locations.

Supplemental Figure S7: Nucleotide diversity (Panel A) and Tajima’s *D* (Panel B) calculated from 58 Sri Lankan *M. bilineata* sampled from three locations.

Supplemental Table S1: Sample metadata of oysters sequenced by DArTseq in this study.

Supplemental File S1: Unfiltered DArTseq genotypes of *Magallana bilineata* samples sequenced in this study.
