# Supplementary figures and images for: A long-read–based de novo assembly of Magallana bilineata for improved tropical oyster aquaculture
Source: G3 (Bethesda). 2025 Oct 19;15(12):jkaf242. doi: 10.1093/g3journal/jkaf242 (PMC12693617; doi:10.1093/g3journal/jkaf242)

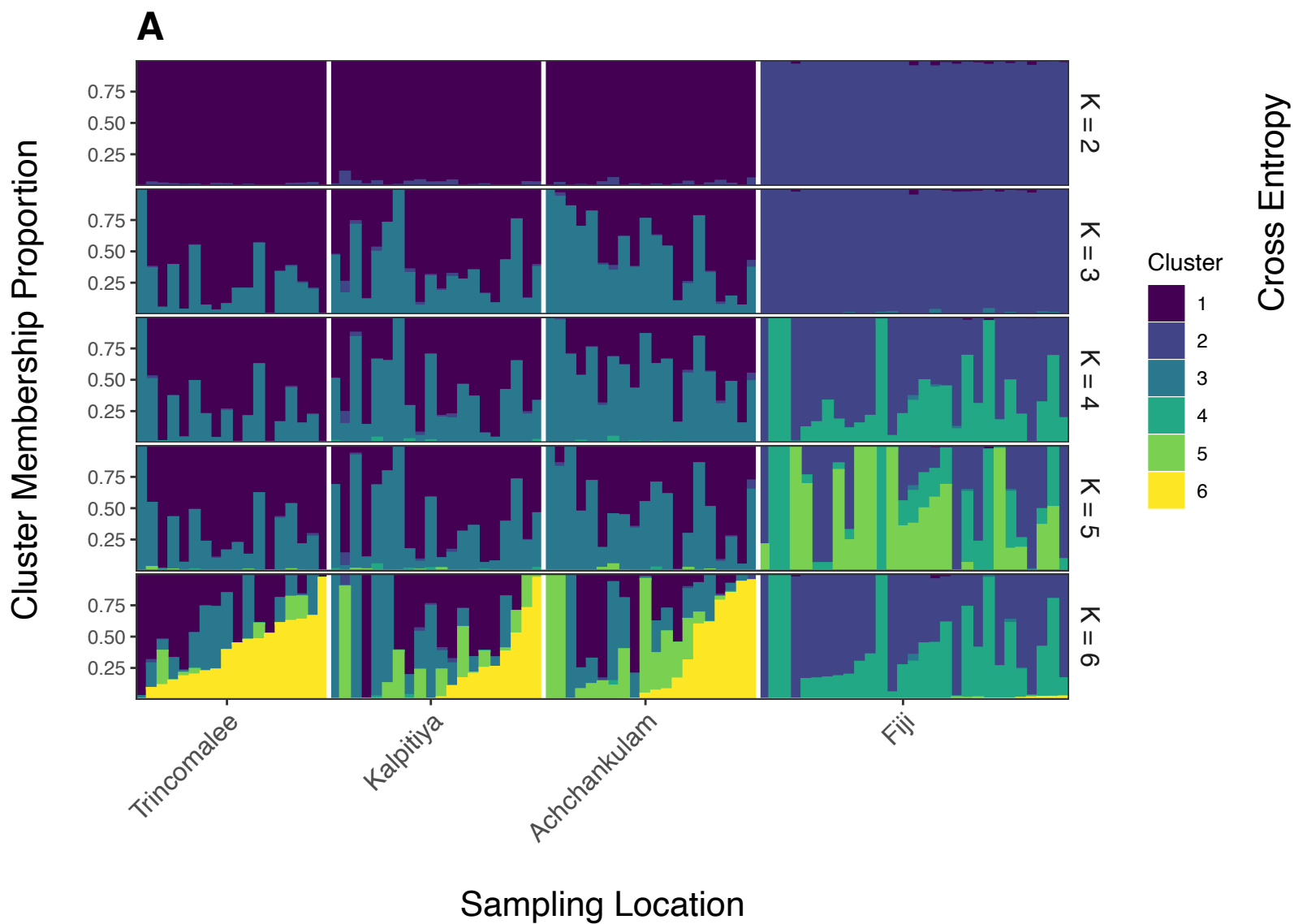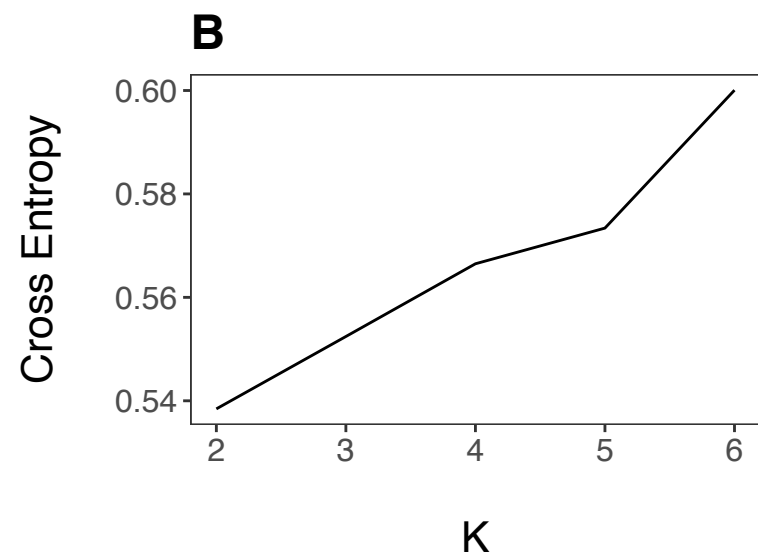

Supplement: jkaf242_Supplementary_Data [file jkaf242_supplementary_data.zip › Supplemental_Figure_S1_G3-2025-406155.pdf]

**A**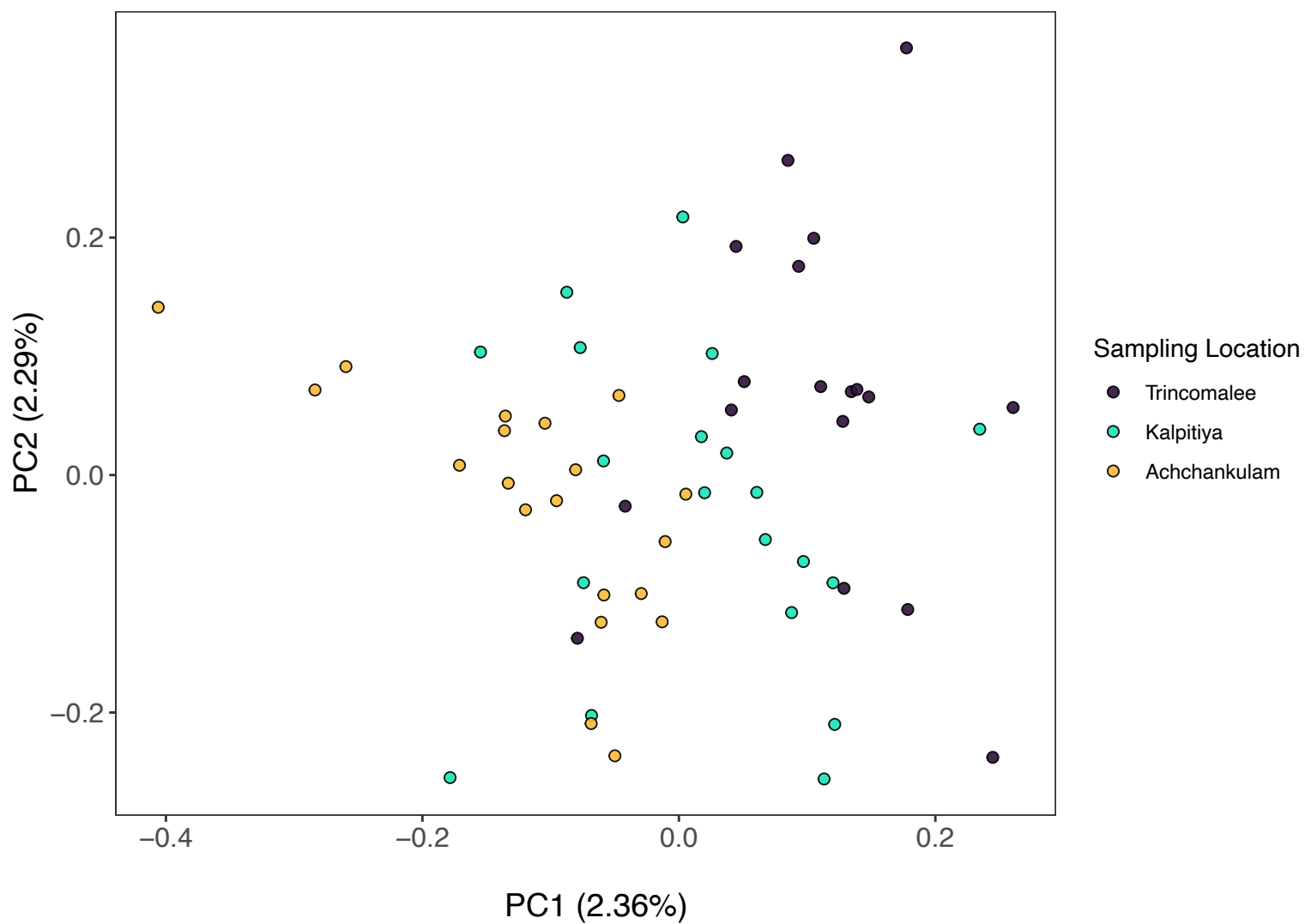**B**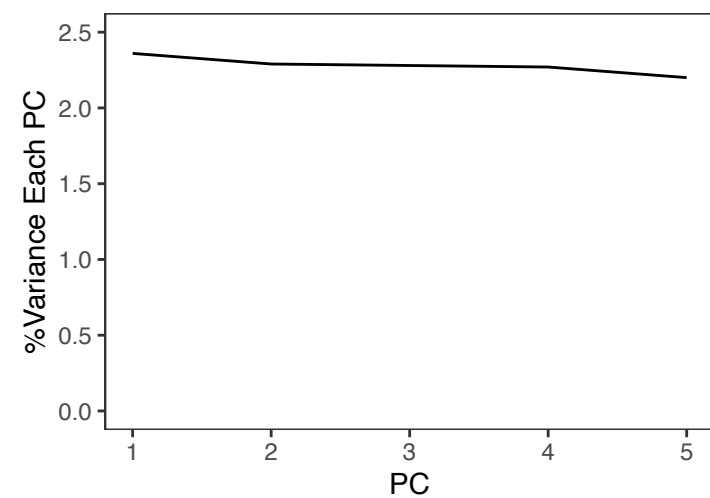**C**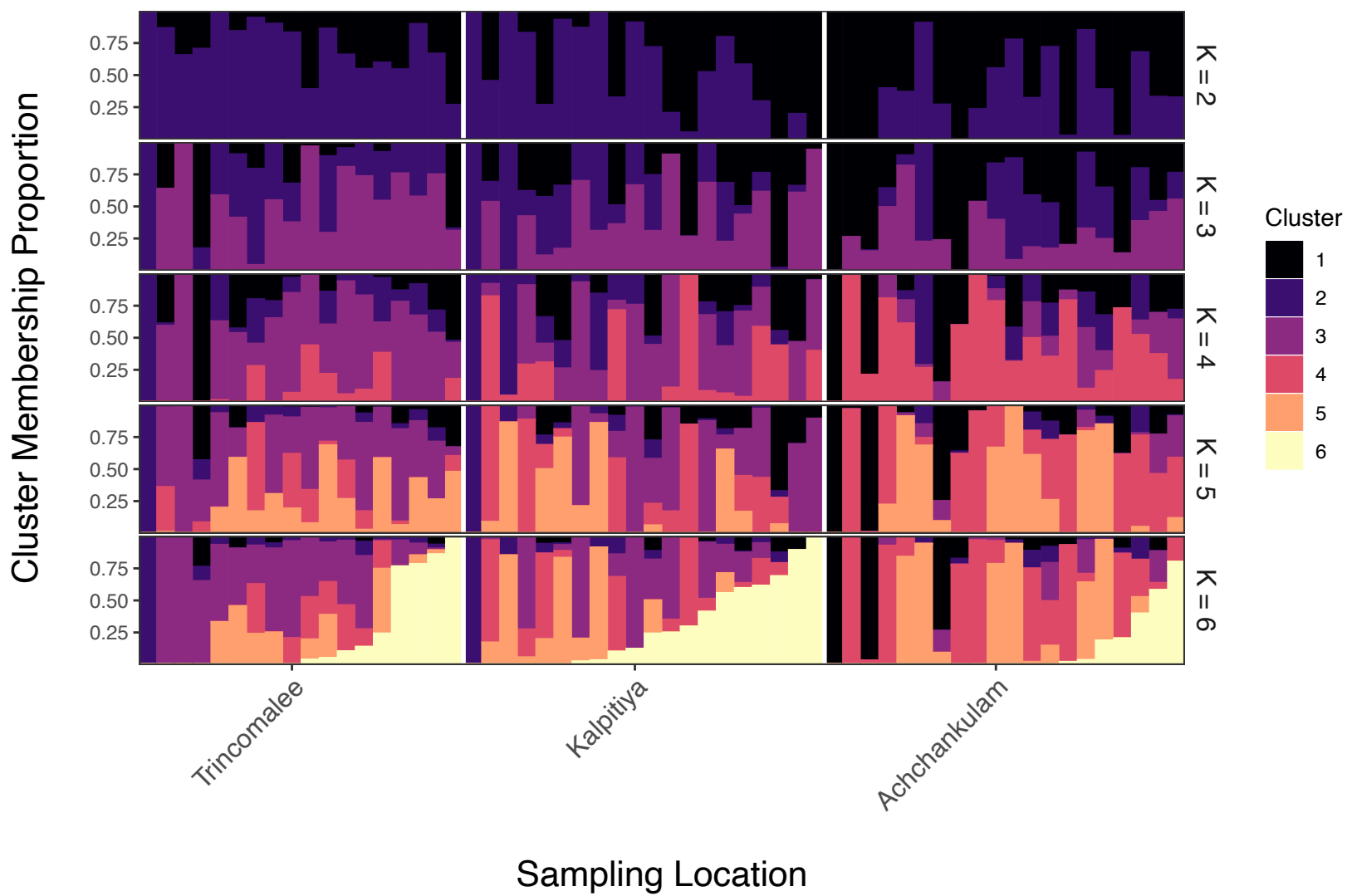**D**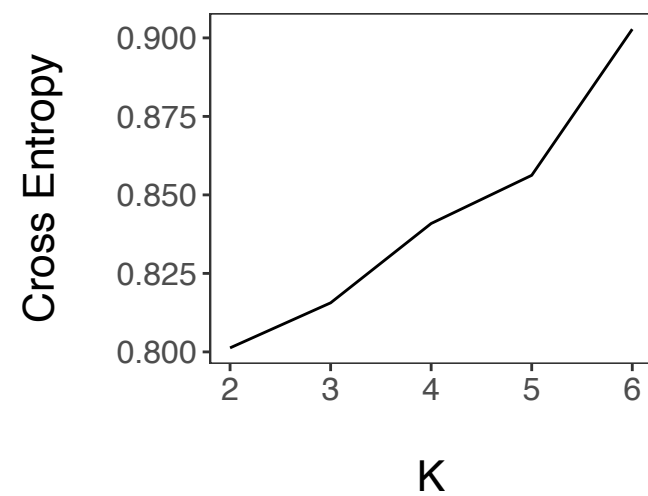

Supplement: jkaf242_Supplementary_Data [file jkaf242_supplementary_data.zip › Supplemental_Figure_S2_G3-2025-406155.pdf]

Population 2

Fiji

Achchankulam

Kalpitiya

Trincomalee

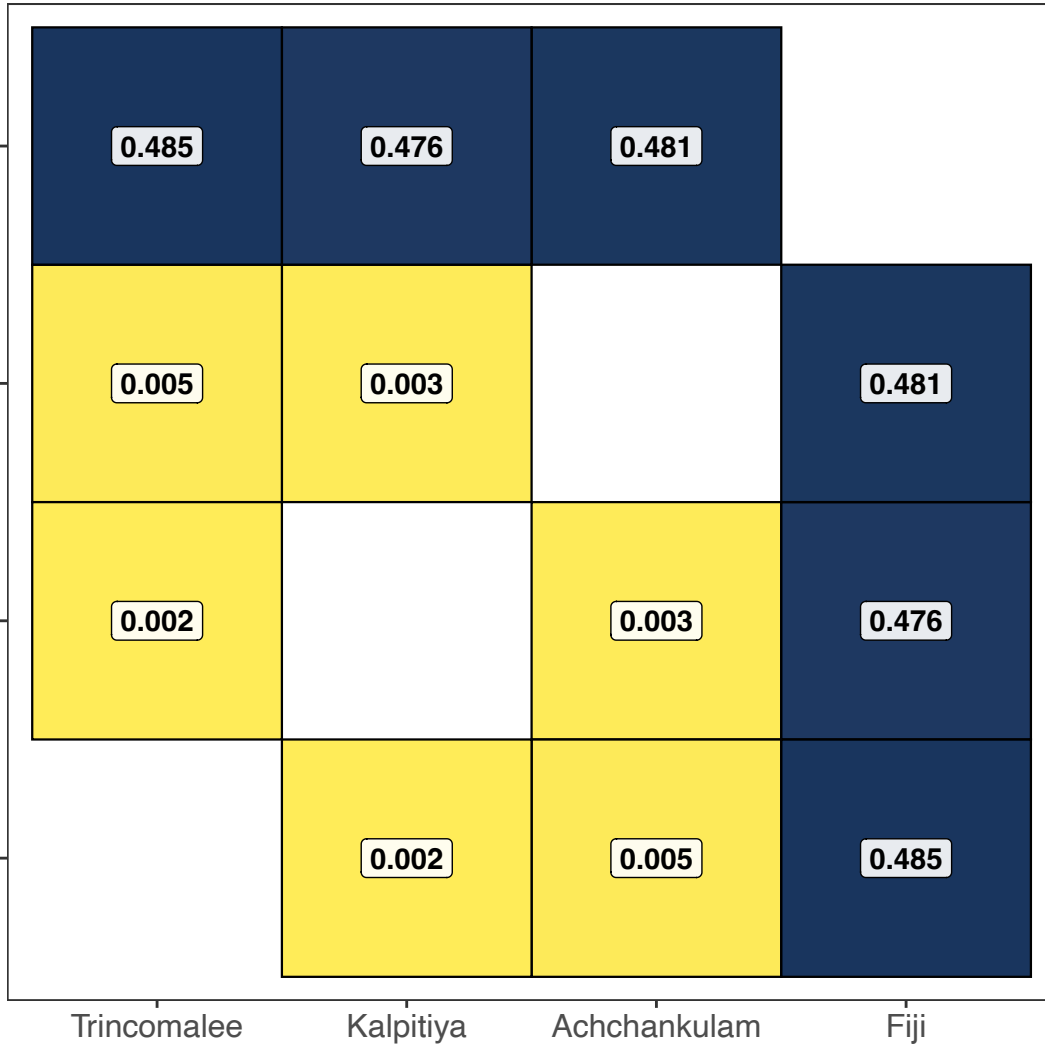

Population 1

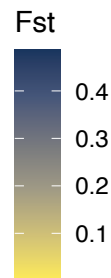

Supplement: jkaf242_Supplementary_Data [file jkaf242_supplementary_data.zip › Supplemental_Figure_S3_G3-2025-406155.pdf]

**A**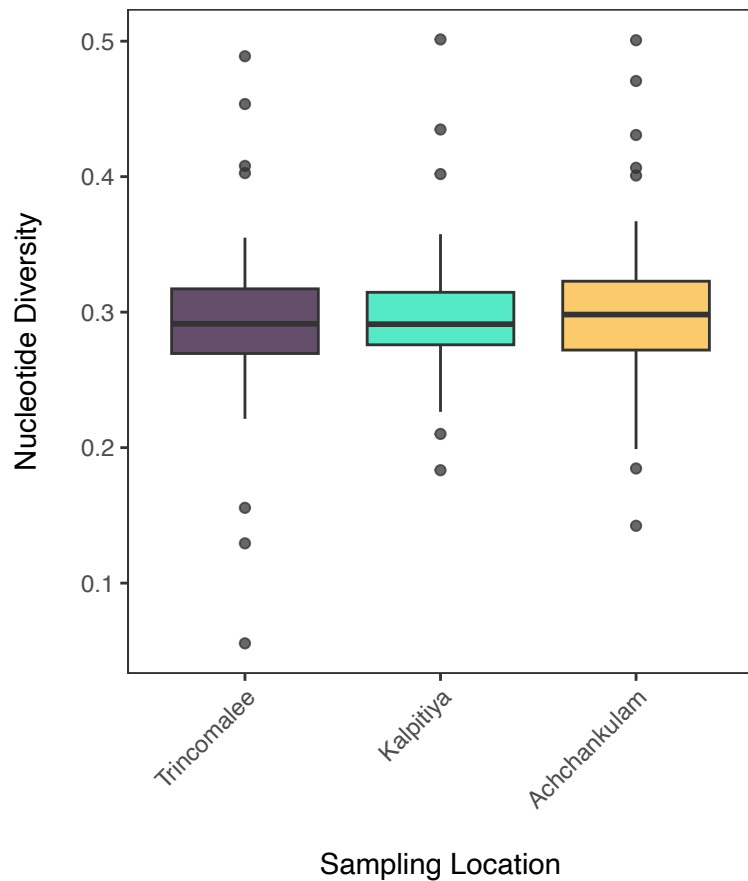**B**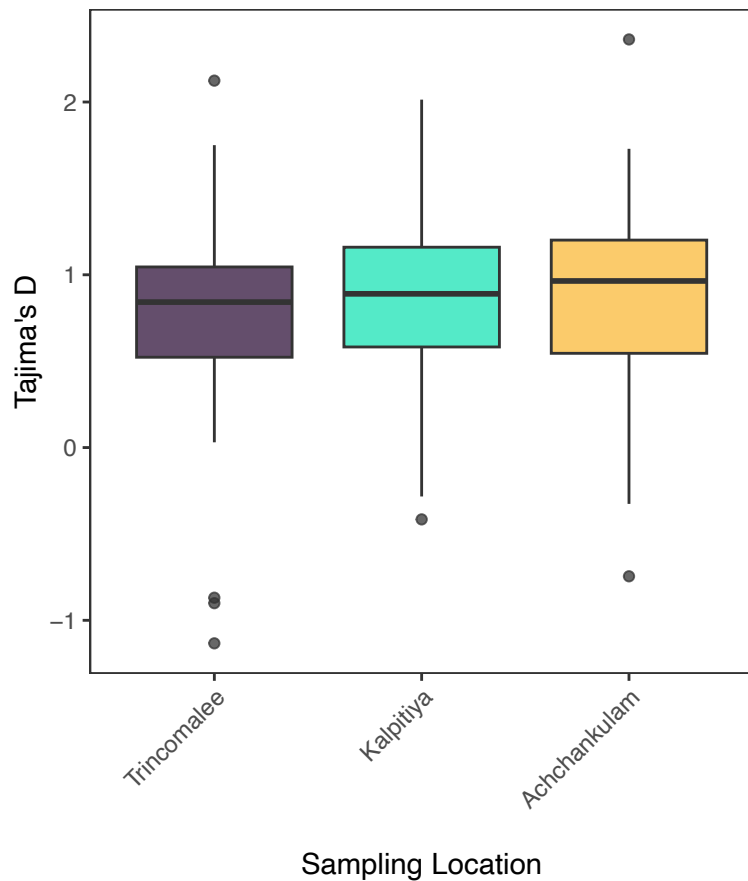

Supplement: jkaf242_Supplementary_Data [file jkaf242_supplementary_data.zip › Supplemental_Figure_S7_G3-2025-406155.pdf]
